# Control of transplant tolerance and intragraft regulatory T cell localization by CCL5

**DOI:** 10.1186/1479-5876-10-S3-P60

**Published:** 2012-11-28

**Authors:** Nahzli Dilek, Nicolas Poirier, Claire Usal, Bernard Martinet, Gilles Blancho, Bernard Vanhove

**Affiliations:** 1 Institute of Transplantation Urology Nephrology, University of Nantes, INSERM UMR 643, Nantes, France Effimune, Nantes, France CHU de Nantes, Nantes, France

## Background

CCL5 (Rantes) is a chemotactic cytokine playing an active role in recruiting leukocytes into inflammatory sites and known to attract Treg cells in solid tumors where they inhibit immune responses.

## Material, methods and results

In rat recipients of kidney allografts where tolerance was induced by costimulation blockade, we observed a 2-fold decrease of plasma CCL5 levels as compared to control syngeneic grafted animals (0.60 ± 0.17 *vs.* 1.17 ± 0.48 ng/ml; p<0.01). Myeloid-derived suppressor cells (MDSC), a major cell type producing CCL5 in blood, also presented a reduced capacity to produce CCL5 in these tolerant animals. In contrast, intragraft levels of CCL5 mRNA and protein were higher in tolerated allografts, establishing an increased graft-to-periphery CCL5 gradient possibly contributing to the recruitment of Treg cells into the graft, as in solid tumor, and leading to the establishment and maintenance of tolerance. To test the hypothesis, we broke the gradient by restoring normal plasma concentrations of CCL5 by implantation of osmotic pumps. This induced a strong reduction of intragraft Treg cells (decrease of 24.40 ± 5.47 fold; p<0.05 by immunohistofluorescence and of Foxp3 mRNA assessed by qRT-PCR), and led to an increase of creatinine and urea concentrations and eventually to kidney graft rejection.

## Conclusions

Our data uncover a novel role of CCL5, possibly controlled by MDSC, in this rat model of kidney transplant tolerance: a graft-to-periphery gradient of CCL5 help recruiting Treg cells into the graft where they maintain tolerance.

